# Adipocyte-Derived Extracellular Vesicles Promote Prostate Cancer Cell Aggressiveness by Enabling Multiple Phenotypic and Metabolic Changes

**DOI:** 10.3390/cells11152388

**Published:** 2022-08-03

**Authors:** Fabrizio Fontana, Martina Anselmi, Emanuela Carollo, Patrizia Sartori, Patrizia Procacci, David Carter, Patrizia Limonta

**Affiliations:** 1 Department of Pharmacological and Biomolecular Sciences, Università degli Studi di Milano, 20133 Milano, Italy; martina.anselmi@unimi.it (M.A.); patrizia.limonta@unimi.it (P.L.); 2 Department of Biological and Medical Sciences, Oxford Brookes University, Oxford OX3 0BP, UK; manu.carollo-2017@brookes.ac.uk (E.C.); dcarter@brookes.ac.uk (D.C.); 3 Department of Biomedical Sciences for Health, Università degli Studi di Milano, 20133 Milano, Italy; patrizia.sartori@unimi.it (P.S.); patrizia.procacci@unimi.it (P.P.)

**Keywords:** prostate cancer, obesity, adipocytes, extracellular vesicles, metastasis, chemoresistance, Warburg effect

## Abstract

Background: In recent decades, obesity has widely emerged as an important risk factor for prostate cancer (PCa). Adipose tissue and PCa cells have been shown to orchestrate a complex interaction network to support tumor growth and evolution; nonetheless, the study of this communication has only been focused on soluble factors, although increasing evidence highlights the key role of extracellular vesicles (EVs) in the modulation of tumor progression. Methods and Results: In the present study, we found that EVs derived from 3T3-L1 adipocytes could affect PC3 and DU145 PCa cell traits, inducing increased proliferation, migration and invasion. Furthermore, conditioning of both PCa cell lines with adipocyte-released EVs resulted in lower sensitivity to docetaxel, with reduced phosphatidylserine externalization and decreased caspase 3 and PARP cleavage. In particular, these alterations were paralleled by an Akt/HIF-1α axis-related Warburg effect, characterized by enhanced glucose consumption, lactate release and ATP production. Conclusions: Collectively, these findings demonstrate that EV-mediated crosstalk exists between adipocytes and PCa, driving tumor aggressiveness.

## 1. Introduction

Prostate cancer (PCa) is the most commonly diagnosed malignancy and the third leading cause of cancer-related deaths among men in western countries [1]. This pathology initially responds to androgen deprivation treatment; however, it often progresses into castration-resistant prostate cancer (CRPC), a condition where tumor cells acquire the ability to escape cell death and become resistant to current strategies, particularly to taxane-based chemotherapy [2,3,4]. 

Interestingly, in the last two decades, a progressive increase in the incidence of PCa has been observed, accompanied by a parallel rise in the prevalence of obesity and metabolic syndrome [5,6,7,8,9]. While the association between obesity and PCa initiation is still a matter of debate, an increasing body of evidence suggests that an obese condition correlates with tumor recurrence and poor prognosis. Various processes have been demonstrated to be involved in the obesity-driven PCa progression, such as hormonal and inflammatory alterations and a deregulated lipid metabolism [5,6,7,8,9,10]. Nonetheless, until now, the study of this dialog has been limited to adipokines and metabolites, although an emerging body of evidence points to the key role of stromal cell-derived extracellular vesicles (EVs) in the regulation of tumor progression. 

Extracellular vesicles are nano-sized particles involved in cell-to-cell communication [11]. In particular, they are responsible for the transfer of proteins, RNAs and miRNAs from the originating cells to both neighboring and distant cells [12]. EVs have been shown to modulate different tumorigenic processes, including cancer proliferation and invasion, as well as tumor drug resistance [13,14]. Moreover, they are implicated in the interactions between tumor cells and their microenvironment, including adipocytes [15,16]. For example, it has been shown that EVs from cancer-associated adipocytes promote breast cancer cell growth via activation of the Hippo signaling pathway and lung cancer metastasis by increasing MMP9 activity through direct MMP3 transfer [17,18]. In addition, adipocyte-derived EVs have been found to carry proteins implicated in fatty acid oxidation (FAO), thus increasing melanoma lipid metabolism and aggressiveness [19,20]. Finally, they have been found to confer chemoresistance to ovarian cancer cells, by transferring miRNA-21 [21]. As mentioned above, it is still unclear how these microvesicles can modulate the adipose tissue-mediated PCa progression, although new findings suggest that adipocyte-associated EVs can enhance PC3 PCa cell invasiveness in vitro [19].

Herein, we shed further light on the molecular mechanisms underlying the dialog between adipose tissue and PCa, with particular emphasis on the role of EVs in modulating tumor cell aggressiveness.

## 2. Materials and Methods

### 2.1. Chemicals

Docetaxel was purchased from Sigma-Aldrich (Milano, Italy).

The primary antibodies ALIX (2171), *p*-Akt (9271), Akt (2938) and HIF-1α (3716) were from Cell Signaling Technology Inc. (Danvers, MA, USA). TSG101 antibody (ab30871) was from Abcam (Cambridge, UK). Hsc70 (13D3) and calnexin (AF18) antibodies were from Thermo Fisher Scientific (Waltham, MA, USA). Cytochrome *c* antibody (sc-13560) was from Santa Cruz Biotechnology Inc. (Santa Cruz, CA, USA). α-tubulin antibody (T6199) was from Sigma-Aldrich. All the antibodies were used at the concentration 1:1000.

Horseradish-peroxidase-conjugated secondary antibody and enhanced chemiluminescence reagents were from Cyanagen (Bologna, Italy).

### 2.2. Cell Lines and Cell Culture

PC3 and DU145 PCa cells were from American Type Culture Collection (ATCC, Manassas, VA, USA), and they were grown in RPMI media containing 7.5% and 5% FBS respectively, glutamine and antibiotics, in humidified atmosphere of 5% CO2/95% air at 37 °C. 3T3-L1 pre-adipocytes were also from ATCC and were grown in DMEM media containing 10% FBS, glutamine and antibiotics. Following resuscitation from freezing in liquid nitrogen, cells were cultured in the appropriate media for 10–12 weeks. Cells were harvested by trypsin-EDTA solution and passaged once/week.

### 2.3. 3T3-L1 Cell Differentiation

3T3-L1 pre-adipocyte differentiation was obtained by replacing regular media with induction media supplemented with 10% FBS and 500 μM 3-isobutyl-1-methylxanthine, 1 μM dexamethasone, 1 μg/mL insulin and 1 μM rosiglitazone. Three days later, the induction media was replaced with DMEM supplemented with 10% FBS and 1 μg/mL insulin, and cells were maintained in culture for additional 4 days. Then, mature adipocytes were cultured in regular media for other 3 days. 

### 2.4. Extracellular Vesicle Extraction

FBS was ultracentrifuged at 120,000× *g* for 16 h; DMEM was then supplemented with EV-depleted bovine serum to obtain EV-depleted media (EDM). Cells in 10-cm dishes were grown for 48 h in EDM. EVs were extracted from this conditioned media by size exclusion chromatography (SEC). Initially, it was centrifuged at 300× *g* for 5 min followed by centrifugation at 16,500× *g* for 20 min at 4 °C. The media was then filtered at 3000× *g* via a Vivaspin PES 100 kDa cutoff filter (Sigma-Aldrich), and 0.5 mL of the sample was applied to a sepharose-based column (Bio-Rad Laboratories, Hercules, CA, USA), using PBS as the eluent. The SEC fractions 7 to 11 (0.5 mL each) were pooled and further filtrated at 3000× *g* via a Vivaspin PES 5 kDa cutoff filter (Sigma-Aldrich), until obtaining 100 µL of sample. When not used immediately after extraction, EVs were stored at −80 °C.

### 2.5. Nanoparticle Tracking Analysis

Nanoparticle tracking analysis (NTA) was performed to determine the EV size and concentration, by using a NanoSight LM10 instrument equipped with the NTA 2.0 analytical software (Malvern Instruments Ltd., Malvern, UK). Five 30 s videos/sample were obtained and used to measure EV mean diameter (nanometres) and concentration (10^8^ mL^−1^). Each sample was run in duplicate.

### 2.6. Transmission Electron Microscopy

For TEM analysis of EVs, 6 μL of vesicles (30 μg/mL) were pipetted into 300 mesh copper grids with Formvar carbon-coated film. After 3 min, excess liquid was removed by blotting, and the grids were stained with 2% aqueous uranyl acetate for 1 min. The grids were then quickly washed in distilled water and drained using filter paper. Finally, they were air dried at room temperature and observed under a Zeiss EM10 electron microscope (Carl Zeiss, Oberkochen, Germany).

### 2.7. Cell Proliferation Assay

PCa cells were seeded (5 × 10^4^ cells/well) in six-well plates. After 48 h, they were treated with adipocyte-derived EVs (30 μg/mL) for 96 h. Cells were then harvested, stained with Trypan blue 0.4% (1:1 *v*/*v*) and counted by Luna automated cell counter (Logos Biosystems, Annandale, VA, USA).

In the case of docetaxel treatment, PCa cells were seeded (5 × 10^4^ cells/dish) in six-well plates, and after 48 h, they were exposed to adipocyte-derived EVs (30 μg/mL) for 24 h and then to docetaxel (10 nM) for 48 h. Adherent (viable) and floating (dead) cells were harvested and counted as above.

### 2.8. Cell Cycle Analysis

PCa cells were seeded (5 × 10^4^ cells/dish) in six-well plates. After 48 h, they were treated with adipocyte-derived EVs (30 μg/mL) for 24 h. Cell cycle analysis was performed by using the Invitrogen™ FxCycle™ PI/RNase Staining Solution (according to manufacturer’s instructions, Thermo Fisher Scientific) and a Novocyte3000 flow cytometer (ACEA Biosciences, San Diego, CA, USA).

### 2.9. Wound Healing Assay

PCa cells were seeded (2 × 10^5^ cells/dish) in six-well plates. After 48 h, a wound healing assay was conducted by scratching the cell layer before EV treatment (30 μg/mL). Wound images were acquired immediately after scratching as well as after 12 and 24 h by using a Zeiss Axiovert 200 microscope (Carl Zeiss) with a 10 × 1.4 objective lens linked to a Coolsnap Es CCD camera (Roper Scientific-Crisel Instruments, Roma, Italy).

### 2.10. Boyden Chamber Assay

PCa cells were seeded (5 × 10^4^ cells/dish) in six-well plates. After 48 h, they were treated with adipocyte-derived EVs (30 μg/mL) for 24 h. Cells were then harvested, resuspended in serum-free culture media and placed in the open-bottom wells of the upper compartment of the Boyden chamber (1 × 10^5^ cells/well). The chemoattractant (FBS 5%) was placed in the lower compartment of the chamber. The two compartments were separated by a polyvinylpyrrolidone-free polycarbonate porous membrane (8 μm pores) precoated with gelatin (0.2 mg/mL in PBS). The chamber was then placed in the incubator for 6 h. After that, cells invading the lower surface of the membrane were fixed, stained with Diff-Quick kit (DADE, Dudingen, Switzerland), and counted in three randomly selected microscope fields.

### 2.11. Annexin V/PI Apoptosis Assay

PCa cells were seeded (5 × 10^4^ cells/well) in six-well plates, and after 48 h, they were exposed to adipocyte-derived EVs (30 μg/mL) for 24 h and then to docetaxel (100 nM) for 48 h. Annexin V/PI apoptosis assay was performed by using the eBioscience™ Annexin V-FITC Apoptosis Detection Kit (Thermo Fisher Scientific) and a Novocyte3000 flow cytometer, as described in [22].

### 2.12. Measurement of Glucose Consumption

PCa cells were seeded (5 × 10^4^ cells/well) in six-well plates. After 48 h, they were treated with adipocyte-derived EVs (30 μg/mL) for 24 h. Glucose consumption was measured by using 2-(N-(7-Nitrobenz-2-oxa-1,3-diazol-4-yl)Amino)-2-Deoxyglucose (100 μM for 30 min, Thermo Fisher Scientific) and a Novocyte3000 flow cytometer, as described in [22].

### 2.13. Measurement of Lactate Synthesis

Lactate synthesis was quantified by using a lactate assay kit (Sigma-Aldrich) and an EnSpire Multimode Plate reader (PerkinElmer, Milano, Italy), as described in [22].

### 2.14. Measurement of ATP Synthesis

ATP synthesis was quantified by using an ATP assay kit (GeneTex, Alton Pkwy Irvine, CA, USA) and an EnSpire Multimode Plate reader, as described in [22].

### 2.15. Western Blot Analysis

Cells were seeded at 5 × 10^4^ cells/well in six-well plates. After each treatment, they were lysed in RIPA buffer. Protein extracts (20 μg) were resolved on SDS-PAGE and transferred to nitrocellulose membranes, which were incubated with the specific primary and horseradish peroxidase-conjugated secondary antibodies. For detection, enhanced chemiluminescence (Westar Etac Ultra 2.0, XLS075,0100, Cyanagen) was used.

### 2.16. Statistical Analysis

Statistical analysis was conducted by using a statistic package (GraphPad Prism5, GraphPad Software San Diego, CA, USA). Data are reported as the mean ± SEM of three independent experiments. *T*-test or one-way analysis of variance (ANOVA) followed by Bonferroni’s test were used to assess the differences between groups. A *p* value < 0.05 was considered statistically significant.

## 3. Results

### 3.1. Characterization of Adipocyte-Derived Extracellular Vesicles

**Figure 1:**
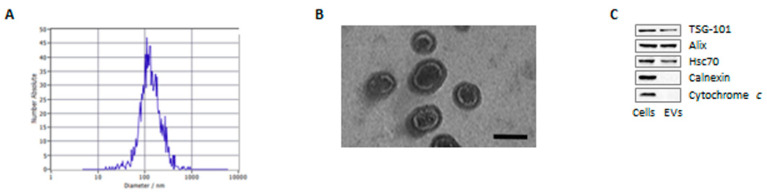
Characterization of adipocyte-derived extracellular vesicles. (**A**) EV size measured by nanoparticle tracking analysis. (**B**) EV morphology visualized by transmission electron microscopy. Scale bar is 100 nm. (**C**) Western blot analysis was performed to investigate the expression levels of TSG101, Alix, Hsc70, calnexin and cytochrome *c* in 3T3-L1 adipocytes and in their EVs. One representative of three experiments performed is shown.

As a first step in assessing the role of EVs in PCa microenvironment, we analyzed the characteristics of adipocyte-derived particles. Vesicles were purified from fully-differentiated 3T3-L1 conditioned media by SEC, and they were found to display the expected size (30–160 nm) and morphology (Figure 1A,B). In addition, they contained various EV hallmarks, namely TSG101, Alix and Hsc70, with no cellular contamination, as indicated by the absence of calnexin and cytochrome *c* (Figure 1C). Overall, these data show that our isolated fractions are enriched in EVs.

### 3.2. Adipocyte-Released Extracellular Vesicles Promote Prostate Cancer Cell Proliferation

**Figure 2:**
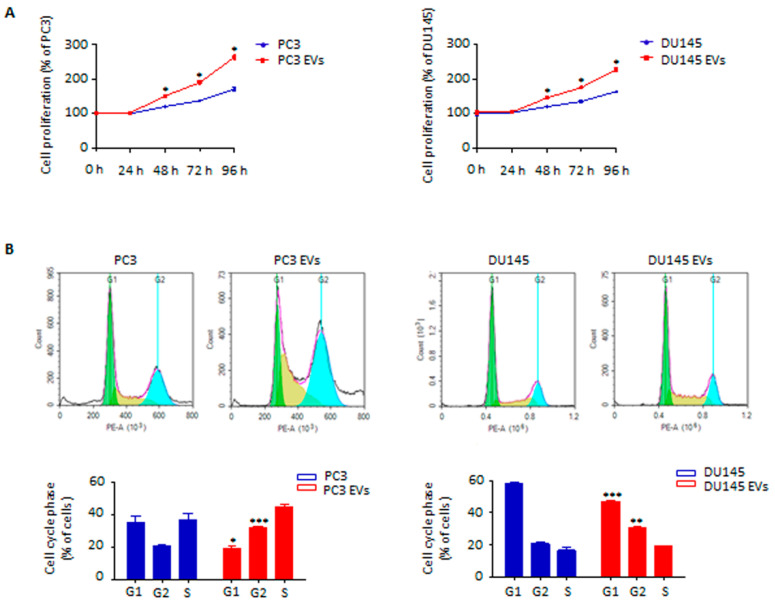
Adipocyte-released EVs promote prostate cancer cell proliferation. (**A**) PC3 and DU145 cells were incubated with adipocyte-released EVs (30 μg/mL) for 96 h. Cell proliferation was then evaluated by Trypan Blue exclusion assay. Each experiment was repeated three times. Data represent mean values ± SEM and were analyzed using a *t*-test. * *p* < 0.05 vs. PC3 or DU145 (control). (**B**) Cells were incubated with adipocyte-released EVs (30 μg/mL) for 24 h. Cell cycle distribution was then evaluated by cytofluorimetric analysis after staining with Invitrogen™ FxCycle™ PI/RNase Staining Solution (according to the manufacturer’s protocol). Each experiment was repeated three times. Data represent mean values ± SEM and were analyzed using a *t*-test. * *p* < 0.05 vs. PC3 or DU145 (control), ** *p* < 0.01 vs. PC3 or DU145 (control), *** *p* < 0.001 vs. PC3 or DU145 (control).

We next hypothesized that adipocyte-released EVs could affect PCa growth. To test this hypothesis, PC3 and DU145 cell lines were treated with vesicles from mature 3T3-L1 cells. Remarkably, this resulted in an increased tumor cell proliferation (Figure 2A). In particular, the number of cells within the S/G2 phase was significantly enhanced after conditioning with stromal particles (Figure 2B). On the other hand, the exposure of PC3 and DU145 cells to EVs from non-differentiated 3T3-L1 pre-adipocytes did not lead to a significant increase in tumor aggressiveness (Figure S1A), indicating that the above effects are selectively induced by adipocyte-derived vesicles. Collectively, these findings highlight that EVs obtained from adipocytes are able to promote PCa cell growth.

### 3.3. Adipocyte-Associated EVs Stimulate Prostate Cancer Cell Migration and Invasion

**Figure 3:**
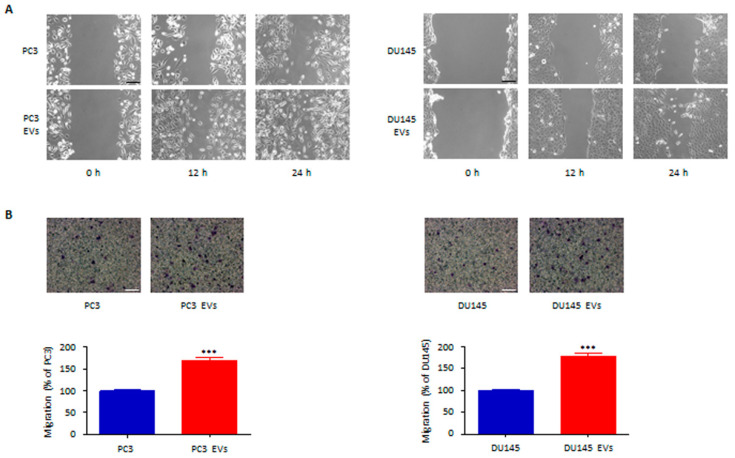
Adipocyte-associated EVs stimulate prostate cancer cell migration and invasion. (**A**) PC3 and DU145 cells were incubated with adipocyte-associated EVs (30 μg/mL) for 24 h. Cell migration was then evaluated by wound healing assay. Each experiment was repeated three times. Scale bar is 200 μm. (**B**) Cells were incubated with adipocyte-associated EVs (30 μg/mL) for 24 h. Cell invasion was then evaluated by Boyden chamber assay. Each experiment was repeated three times. Scale bar is 100 μm. Data represent mean values ± SEM and were analyzed using a *t*-test. *** *p* < 0.001 vs. PC3 or DU145 (control).

We then analyzed the pro-metastatic activity of the EVs from adipocytes. Notably, stromal vesicles were found to drive both PC3 and DU145 cell migration and invasion (Figure 3A,B). It should be noted that incubation of tumor cells with pre-adipocyte-associated vesicles was not followed by any change in PCa cell metastatic potential (Figure S1B), further supporting the specificity of the EV-mediated cross-talk between adipocytes and PCa. Taken together, these results evidence the pro-invasive function of adipocyte-derived EVs in PCa microenvironment.

### 3.4. Adipocyte-Secreted Extracellular Vesicles Enhance Prostate Cancer Cell Chemoresistance

**Figure 4:**
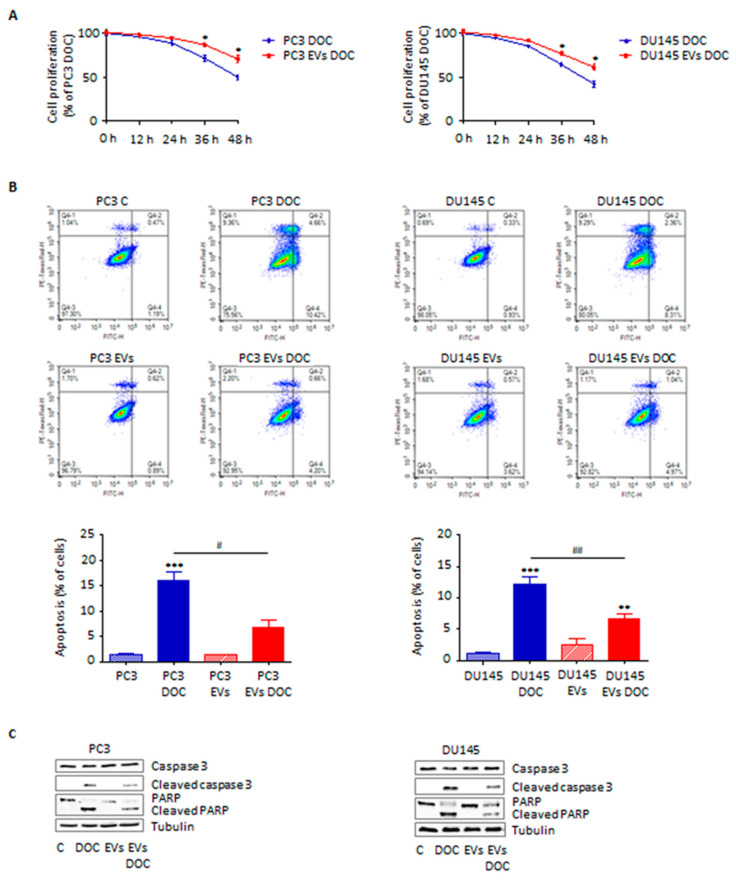
Adipocyte-secreted EVs enhance prostate cancer cell chemoresistance. (**A**) PC3 and DU145 cells were pre-treated with adipocyte-secreted EVs (30 μg/mL) for 24 h and then treated with docetaxel (100 nM) for 48 h. Cell proliferation was then evaluated by Trypan Blue exclusion assay. Each experiment was repeated three times. Data represent mean values ± SEM and were analyzed using a *t*-test. * *p* < 0.05 vs. PC3 DOC or DU145 DOC (control). (**B**) Cells were pre-treated with adipocyte-secreted EVs (30 μg/mL) for 24 h and then treated with docetaxel (100 nM) for 48 h. Apoptotic rate was then evaluated by cytofluorimetric analysis after staining with eBioscience™ Annexin V-FITC Apoptosis Detection Kit (according to the manufacturer’s protocol). One representative of three experiments performed is shown. Data represent mean values ± SEM and were analyzed using a Bonferroni’s test after one-way analysis of variance. ** *p* < 0.01 vs. PC3 or DU145, *** *p* < 0.001 vs. PC3 or DU145, *^#^ p* < 0.05 vs. PC3 DOC or DU145 DOC, *^##^ p* < 0.01 vs. PC3 DOC or DU145 DOC. (**C**) After adipocyte-secreted EV pre-treatment (30 μg/mL, 24 h) and docetaxel treatment (100 nM, 48 h), Western blot analysis was performed to investigate the expression levels of cleaved caspase 3 and PARP. Tubulin expression was evaluated as a loading control. One representative of three experiments performed is shown.

EVs have recently emerged as crucial modulators of cancer drug resistance [23]. Herein, we demonstrated that adipocyte-secreted particles can promote PC3 and DU145 cell survival to docetaxel treatment (Figure 4A), by decreasing phosphatidylserine externalization and reducing caspase 3 and PARP activation (Figure 4B,C). Once again, vesicles from pre-adipocytes were not able to exert a significant pro-survival activity (Figure S1C), confirming that adipocyte-released EVs are specifically responsible for PCa progression, including development of chemoresistance.

### 3.5. Extracellular Vesicles from Adipocytes Reprogram Prostate Cancer Cell Glucose Metabolism

**Figure 5:**
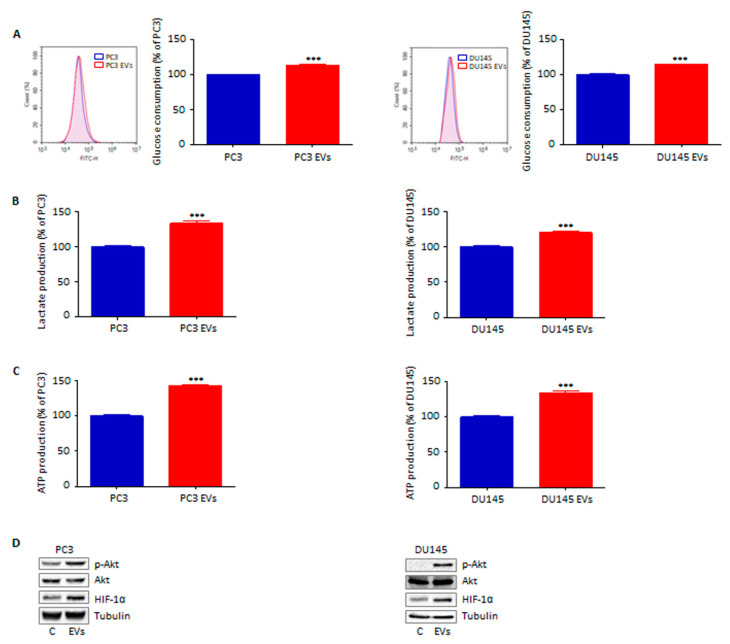
Extracellular vesicles from adipocytes reprogram prostate cancer cell metabolism. (**A**) PC3 and DU145 cells were incubated with adipocyte-derived EVs (30 μg/mL) for 24 h. Glucose consumption was then evaluated by cytofluorimetric analysis after staining with 2-(N-(7-Nitrobenz-2-oxa-1,3-diazol-4-yl)amino)-2-Deoxyglucose (100 μM) for 30 min. Each experiment was repeated three times. Data represent mean values ± SEM and were analyzed using a *t*-test. *** *p* < 0.001 vs. PC3 or DU145 (control). (**B**) Cells were incubated with adipocyte-derived EVs (30 μg/mL) for 24 h. Lactate production was then evaluated by using a lactate assay kit (according to the manufacturer’s protocol). Each experiment was repeated three times. Data represent mean values ± SEM and were analyzed using a *t*-test. *** *p* < 0.001 vs. PC3 or DU145 (control). (**C**) Cells were incubated with adipocyte-derived EVs (30 μg/mL) for 24 h. ATP synthesis was then evaluated by using an ATP assay kit (according to the manufacturer’s protocol). Each experiment was repeated three times. Data represent mean values ± SEM and were analyzed using a *t*-test. *** *p* < 0.001 vs. PC3 or DU145 (control). (**D**) After adipocyte-derived EV treatment (30 μg/mL, 24 h), Western blot analysis was performed to investigate the expression levels of *p*-Akt and HIF-1α in PC3 and DU145 cells. Tubulin expression was evaluated as a loading control. One representative of three experiments performed is shown.

To further elucidate the features of the peculiar phenotype elicited by adipocyte-derived EVs, changes in tumor cell metabolism after vesicular conditioning were investigated. Interestingly, exposure of both PC3 and DU145 cell lines to the particles isolated from 3T3-L1 cell media led to a metabolic switch towards a fast-glycolytic state, characterized by increased glucose consumption, lactate production and ATP synthesis (Figure 5A–C). Importantly, this was accompanied by Akt phosphorylation and subsequent HIF-1α stabilization (Figure 5D), a molecular cascade that is commonly associated with the well-known Warburg effect [24]. As expected, these metabolic alterations were not observed in PCa cells treated with particles from pre-adipocytes (Figure S1D,E). Based on these observations, we can conclude that EVs from adipose cells can effectively rewire PCa glucose metabolism.

## 4. Discussion

It is now widely accepted that a correlation exists between obesity and PCa progression. In fact, increased tumor recurrence and lower overall survival have been observed in obese patients with respect to normal-weight men [5,6,7]. Likewise, periprostatic adipose tissue has been reported to directly influence PCa development by sustaining its growth and metastasis [10,25]. In particular, the dysfunctional adipose mass in overweight patients is primarily responsible for the production of adipokines, growth factors and hormones that are often deregulated and can potentially contribute to tumorigenesis [5,6,7,10,23]. Conversely, little is known about the involvement of adipocyte-secreted EVs in PCa evolution.

Here, we examined the effects of 3T3-L1-derived vesicles on PC3 and DU145 cell aggressiveness, in order to clarify the role of these particles in the modulation of the interactions between the two cell types. Of course, the present study has certain limitations that should be mentioned. For instance, despite being extensively used as a convenient adipocyte model, 3T3-L1 cell line cannot fully recapitulate the characteristics of human primary adipose cells. Additionally, our experiments have only been conducted in vitro; thereby, in vivo studies are a logical next step. Taking these premises into consideration, our results might offer a basis for further research into the role of adipocyte-derived EVs in PCa progression.

First, we found that EVs released from adipocytes mediated a significant pro-tumor activity on PCa cells by stimulating their proliferation. Furthermore, they were shown to significantly enhance cancer cell migration and invasion. This is in agreement with recent reports highlighting the ability of adipocyte-associated EVs to promote cell growth and metastasis in different malignancies, such as breast, hepatocellular and lung carcinoma [17,18,26,27,28]. On the other hand, the above evidence suggests that the complex communication network occurring between PCa and its adipose stroma cannot only be orchestrated by the exchange of specific molecules (such as leptin and TGF-β [25,29]) but also by the vesicular transfer of aggressive tumor traits.

Then, we demonstrated that conditioning with vesicles from 3T3-L1 cells resulted in a significant reduction in PCa sensitivity to chemotherapy; as expected, this was paralleled by a delayed phosphatidylserine externalization on tumor cell membrane as well as by decreased caspase 3 and PARP cleavage. These results are consistent with previous findings demonstrating the fundamental role of adipose-tissue-related particles in determining the emergence of drug resistance in various tumors, such as liver and ovarian cancer [21,27]. Regarding PCa, IGF-1 secreted by periprostatic adipose mass was recently shown to reduce malignant cell susceptibility to docetaxel [30]; our findings not only confirm the ability of adipocytes to modulate cancer chemoresistance but also open the possibility that other adipocyte-released insoluble factors are crucially involved in the development of treatment-unresponsive carcinomas.

In recent years, the characterization of the metabolic interplay between adipocytes and tumor cells has gained wide attention in the study of cancer progression, with a growing body of research indicating that adipocyte-derived fatty acids can directly affect tumor cell metabolic plasticity [31]. Intriguingly, EVs produced by adipose tissue have also been reported to transfer lipids to cancer cells, fueling both tumor proliferation and spread through rapid fatty acid oxidation and energy production [20]. Nonetheless, the role of adipocytes in the emergence of the so-called Warburg effect remains to be elucidated, with a limited number of studies, thus far, investigating the ability of an adipose tissue-enriched environment to sustain cancer glucose metabolism [32,33]. Here, we reported that adipocytes could secrete EVs able to trigger a fast glycolytic flux, characterized by increased glucose uptake, lactate release and ATP synthesis, in PCa cells. In particular, Akt activation and the consequent HIF-1α stabilization, key mechanisms regulating aerobic glycolysis [24], were observed. Notably, this molecular signature is commonly associated with hyperproliferation, metastasis and chemoresistance in PCa [34,35,36,37], suggesting a deep correlation between these metabolic features and the above EV-mediated pro-tumor effects. It should also be underlined that, unlike PTEN-wild type DU145 cell line, PTEN-null PC3 cells heavily rely on glucose consumption for their energetic needs [22,38], presumably explaining their higher susceptibility to EV treatment, particularly in terms of tumor growth. Taken together, these data corroborate the fundamental function of adipocytes in reprogramming tumor metabolism, highlighting for the first time the ability of stromal vesicles to induce a switch towards a glucose-dependent phenotype in PCa. Whether fatty acids contained in the EVs from adipose cells can complement this pathway remains to be addressed; however, given the inability of 3T3-L1 pre-adipocyte-released particles to affect cancer cell behavior, it can be reasonably hypothesized that vesicular lipids may be involved in tumor metabolic rewiring. 

In summary, these results further clarify the mechanisms underlying the crosstalk between adipose tissue and PCa, indicating that adipocyte-released EVs exert a potent tumor-promoting activity by boosting cancer growth, invasion, drug resistance and glucose metabolism.

## Supplementary Materials

The following supporting information can be downloaded at: https://www.mdpi.com/article/10.3390/cells11152388/s1, Figure S1: Extracellular vesicles from pre-adipocytes do not have any effect on prostate cancer cells.
