# Supplementary material for: Adipocyte-Derived Extracellular Vesicles Promote Prostate Cancer Cell Aggressiveness by Enabling Multiple Phenotypic and Metabolic Changes
Source: Cells. 2022 Aug 3;11(15):2388. doi: 10.3390/cells11152388 (PMC9368412; doi:10.3390/cells11152388)
Supplement: Supplementary file 1 [file cells-11-02388-s001.zip › cells-1830615-supplementary.pdf]

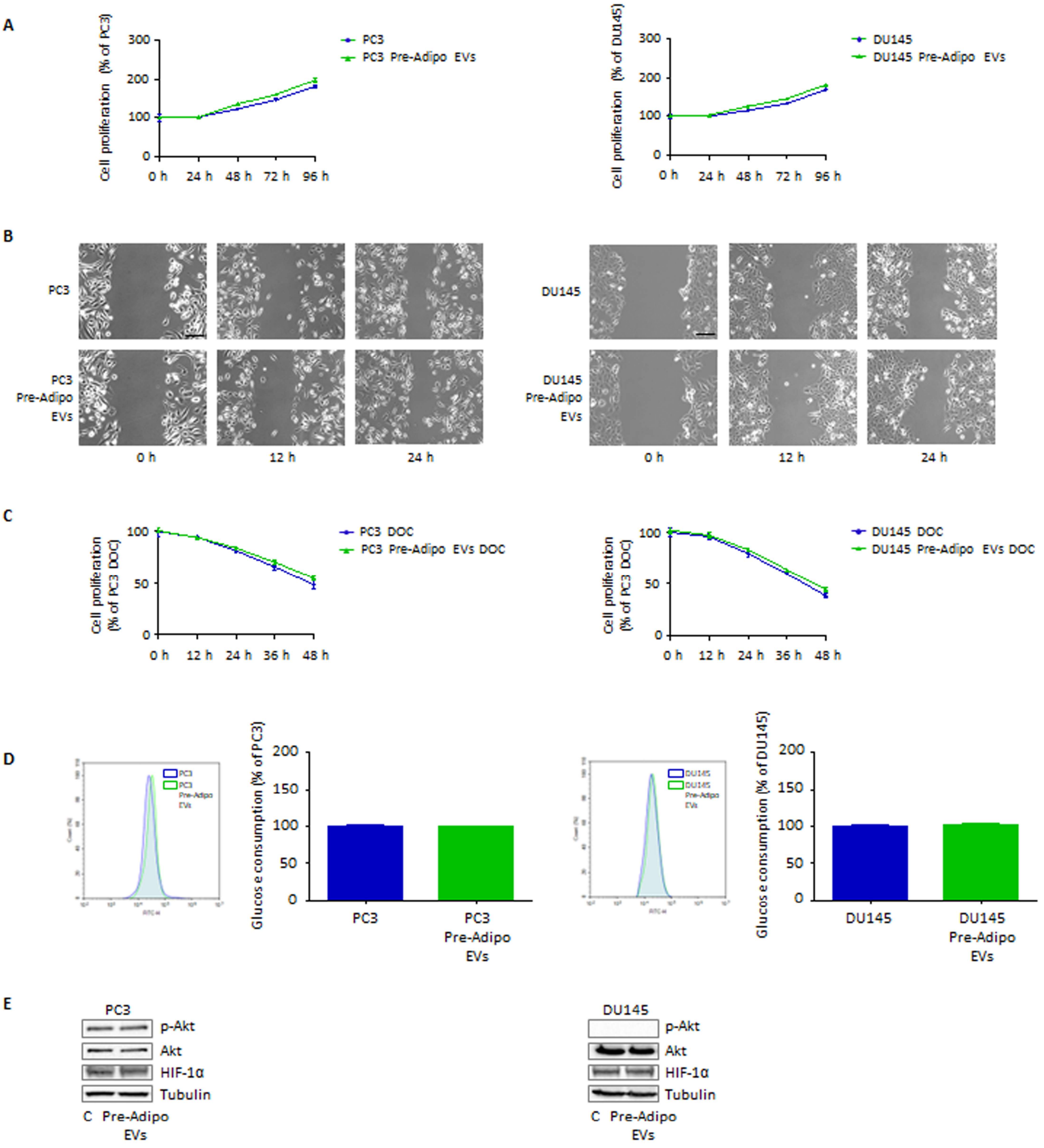

Figure S1: Extracellular vesicles from pre-adipocytes do not have any effect on prostate cancer cells. (**A**) PC3 and DU145 cells were incubated with pre-adipocyte-released EVs (30 µg/mL) for 96 hours. Cell proliferation was then evaluated by Trypan Blue exclusion assay. Each experiment was repeated three times. Data represent mean values ± SEM and were analyzed using a t-test. (**B**) Cells were incubated with pre-adipocyte-associated EVs (30 µg/mL) for 24 hours. Cell migration was then evaluated by wound healing assay. Each experiment was repeated three times. Scale bar is 200 µm. (**C**) Cells were pre-treated with pre-adipocyte-secreted EVs (30 µg/mL) for 24 hours and then treated with docetaxel (100 nM) for 48 hours. Cell proliferation was then evaluated by Trypan Blue exclusion assay. Each experiment was repeated three times. Data represent mean values ± SEM and were analyzed using a t-test. (**D**) Cells were incubated with pre-adipocyte-derived EVs (30 µg/mL) for 24 hours. Glucose consumption was then evaluated by cytofluorimetric analysis after staining with 2-(N-(7-Nitrobenz-2-oxa-1,3-diazol-4-yl)Amino)-2-Deoxyglucose

(100 μM) for 30 min. Each experiment was repeated three times. Data represent mean values ± SEM and were analyzed using a t-test. (**E**) After pre-adipocyte-derived EV treatment (30 μg/mL, 24 hours), Western blot analysis was performed to investigate the expression levels of *p*-Akt and HIF-1α in PC3 and DU145 cells. Tubulin expression was evaluated as a loading control. One representative of three experiments performed is shown.
